# Immune regulation in renal inflammation

**DOI:** 10.1007/s00441-020-03351-1

**Published:** 2021-01-26

**Authors:** Katrin Neumann, Gisa Tiegs

**Affiliations:** 1 Institute of Experimental Immunology and Hepatology, University Medical Center Hamburg-Eppendorf, Hamburg, Germany; 2 Hamburg Center for Translational Immunology, University Medical Center Hamburg-Eppendorf, Hamburg, Germany

**Keywords:** Immune regulation, Immune-mediated glomerulonephritis, Nephrotoxicity, Regulatory T cells, ILC2

## Abstract

Renal inflammation, induced by autoantigen recognition or toxic drugs, leads to renal tissue injury and decline in kidney function. Recent studies have demonstrated the crucial role for regulatory T cells in suppressing pathogenic adaptive but also innate immune responses in the inflamed kidney. However, there is also evidence for other immune cell populations with immunosuppressive function in renal inflammation. This review summarizes mechanisms of immune cell regulation in immune-mediated glomerulonephritis and acute and chronic nephrotoxicity.

## Introduction

Renal inflammation contributes to a multitude of acute and chronic kidney diseases. These include autoimmune kidney diseases such as several forms of glomerulonephritis (GN), which typically result from an overshooting adaptive immune response to autoantigens. However, also more toxic insults, such as ischemia–reperfusion injury (IRI) and cisplatin or adriamycin nephrotoxicity, seem to be mediated by cells of the innate and adaptive immune system. Immunity and inflammation are controlled by immune regulatory mechanisms in order to restore homeostasis. Intriguingly, immune regulation is also elicited in response to IRI or nephrotoxicity induced by cytostatic agents (Sharma and Kinsey 2018). In contrast, autoimmunity results from dysfunction of cells that mediate immunoregulation (Sakaguchi et al. 2008). These cells, originally described to suppress autoimmunity, are named regulatory T cells (Tregs) and represent a subset of CD4^+^ T helper (Th) cells expressing the forkhead box P3 (Foxp3) transcription factor as well as high levels of the interleukin (IL)-2 high-affinity receptor IL-2Rα or CD25 (Sakaguchi et al. 2008). There are two main subsets of Tregs depending on their place of origin. Natural Tregs (nTregs) develop in the thymus by recognition of self-antigens presented by major histocompatibility complex class II (MHCII) molecules in a process called positive selection. In the periphery, nTregs regulate activation of effector T cells recognizing self-antigens and play a crucial role in induction of tolerance against foreign antigens derived from commensal bacteria or food. In contrast, induced Tregs (iTregs) are generated in the periphery by differentiation of naïve CD4^+^ Foxp3^−^ T cells into CD4^+^ Foxp3^+^ Tregs during antigenic stimulation in the presence of IL-2 and transforming growth factor β (TGF β) (Sakaguchi et al. 2008).

In patients, several autoimmune GNs have been associated with aberrant Treg responses, yet displaying heterogeneous mechanisms of dysregulation of immunosuppression with respect to the particular disease. For example, in autoimmune anti-glomerular basement membrane (anti-GBM) GN or Goodpasture’s disease, where autoimmunity develops against the non-collagenous domain of the α3-chain of type IV collagen (α3[IV]NC1), antigen-specific regulatory CD4^+^ CD25^hi^ T cells, capable of suppressing Th1 responses, were absent during active disease but significantly elevated during remission and might be responsible for the rarity of relapses in anti-GBM disease (Salama et al. 2003). Moreover, anti-neutrophil cytoplasmic antibody (ANCA)-associated vasculitis, which can be linked with rapidly progressive glomerulonephritis, was described to correlate with increased numbers of Tregs during remission (Morgan et al. 2010). However, Tregs from ANCA patients seemed to possess decreased suppressive function (Morgan et al. 2010; Rimbert et al. 2011; Free et al. 2013). Systemic lupus erythematosus (SLE) affects multiple organs including lung and kidney. Lupus nephritis has been identified as a major risk factor for a poor prognosis of SLE (Bagavant and Fu 2009). Conflicting results have been shown regarding Treg numbers in SLE patients. While most of the studies reported a decreased proportion of Tregs, others described normal or even increased rates (reviewed in Miyara et al. 2011). Reduced Treg numbers have been positively correlated with disease activity in SLE patients and were associated with an impaired capacity of T cells to produce IL-2 (Humrich et al. 2010; Alcocer-Varela and Alarcon-Segovia 1982; Von Spee-Mayer et al. 2016), which is indispensable for peripheral Treg survival and expansion (Fontenot et al. 2005; D’Cruz and Klein 2005; Setoguchi et al. 2005). Based on these observations, clinical studies on low-dose IL-2 therapy in SLE patients were initiated. Interestingly, low-dose IL-2 expanded nTregs expressing high levels of CD25, retained their suppressive capacity and reduced disease activity in SLE patients (Von Spee-Mayer et al. 2016; He et al. 2016). Finally, reduced Treg frequencies were also shown in patients with immunoglobulin (Ig)A nephropathy (Jin et al. 2018; Lin et al. 2012; Yang et al. 2017), the most frequent cause of primary GN. Here, altered expression of microRNAs (miR) has been associated with Treg deficiency. While elevated expression of miR-133a/133b might prevent FOXP3 translation and thus, Treg differentiation (Jin et al. 2018), miR-155 deficiency might inhibit maturation of Tregs in patients with IgA nephropathy (Yang et al. 2017).

However, most of the studies analyzing immune regulatory mechanisms in renal inflammation were conducted in animal models and therefore, will be summarized here.

## Immune regulation of immune-mediated GN by regulatory T cells

### Nephrotoxic nephritis

A multitude of studies regarding immune regulation in GN have been conducted in the model of rapidly progressive or crescentic GN induced by antibodies raised in sheep or rabbit against protein preparations of the murine glomerular basement membrane (GBM). This model, also referred to as nephrotoxic nephritis (NTN), is not a model of autoimmune GN since glomerular injury develops in response to deposited sheep or rabbit anti-mouse GBM antibodies and not to an endogenous autoantigen. However, NTN is mediated by Th1 and Th17 responses (Kurts et al. 2013), thereby representing a paradigm for immune-mediated GN that allows to study cellular and molecular mechanisms, which activate and regulate immune responses during GN.

**Fig. 1:**
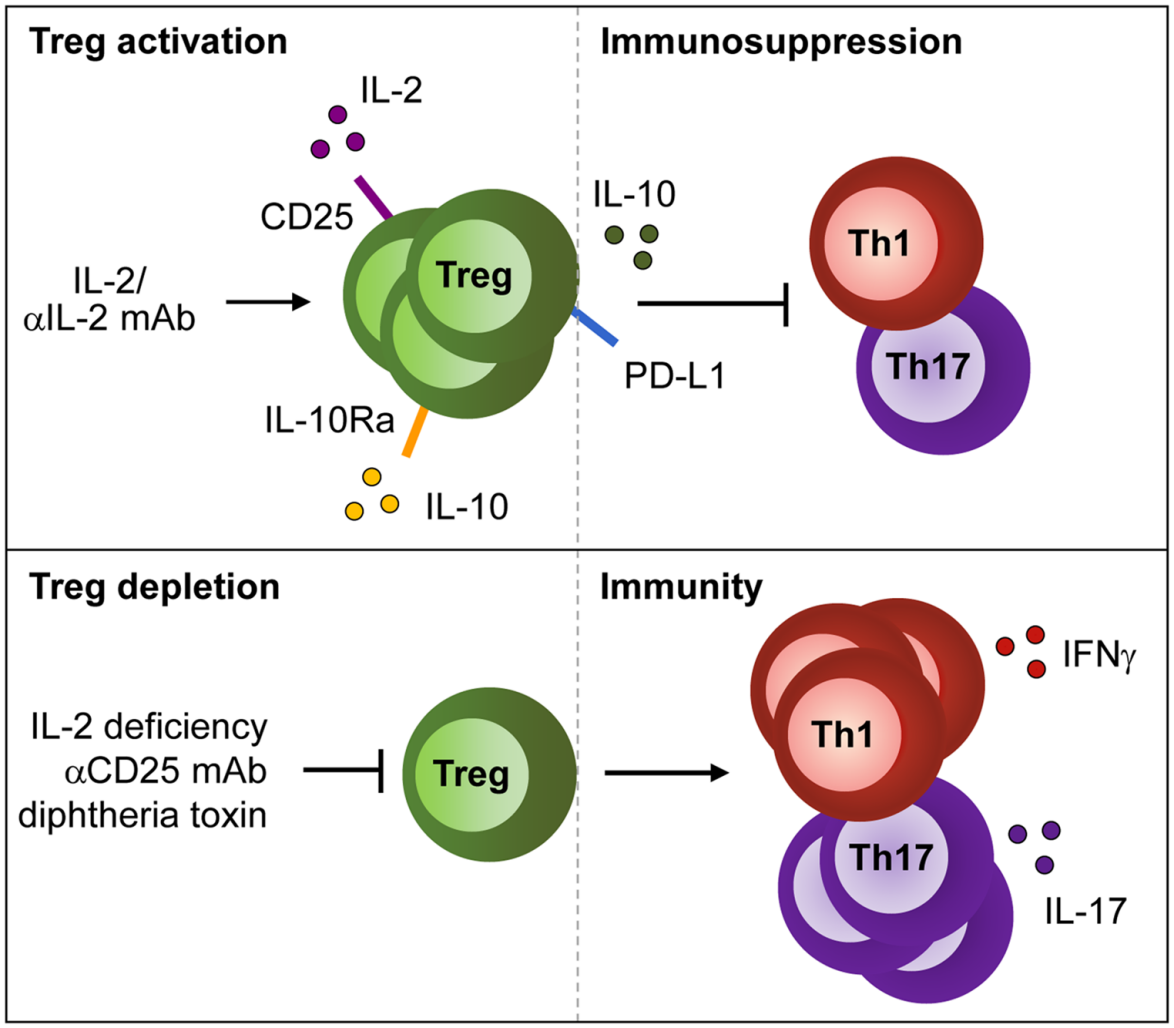
Mechanisms of Treg activation and depletion in immune-mediated GN. Treg activation and function is supported by IL-2, IL-10 and the IL-2/anti-IL-2 mAb complex in immune-mediated GN thereby suppressing the inflammatory Th1 and Th17 response. In contrast, Treg depletion due to IL-2 deficiency or blockage of CD25 increases Th1- and Th17-mediated immunity

Histological analysis of kidneys from nephritic mice revealed crescent formation in glomeruli within 7 days following NTN induction along with infiltration of monocytes/macrophages and T cells. Renal dendritic cells (DCs) have been described as tissue-resident professional antigen-presenting cells (APCs), which are located in the tubulointerstitium (Krüger et al. 2004). They acquire a pathogenic phenotype during NTN and trigger inflammatory CD4^+^ T cell activation, thereby driving pathology of GN (Hochheiser et al. 2011). A pathogenic role of CD4^+^ T cells in NTN was first demonstrated in CD4-deficient mice (Tipping et al. 1998) and in rats after CD4^+^ T cell depletion (Huang et al. 1994). CD4^+^ T cell activation resulted in establishment of renal Th1 (Kitching et al. 1999a, 1999b; Phoon et al. 2008) and Th17 immune responses (Paust et al. 2009; Steinmetz et al. 2011; Odobasic et al. 2011), which aggravated NTN. Adoptive transfer experiments with CD4^+^ CD25^+^ regulatory T cells were the first to show a protective role of Tregs in the NTN model. Transfer of these cells decreased proteinuria and crescent formation, reduced renal infiltration of T cells and macrophages and suppressed the Th1 response (Wolf et al. 2005). Later, two independent studies reported an immunosuppressive role of endogenous Foxp3^+^ Tregs during NTN by using depletion of regulatory T cell (DEREG) mice that express the diphtheria toxin receptor under control of the Foxp3 promoter, thereby allowing selective depletion of Foxp3^+^ Tregs by diphtheria toxin injection (Ooi et al. 2011; Paust et al. 2011). Interestingly, renal inflammation provoked a continuous increase of Tregs over time in the tubulointerstitium and periglomerular space. Moreover, Tregs from nephritic mice were more suppressive in vitro than those from naïve animals (Ooi et al. 2011). Treg depletion resulted in an aggravated course of GN and an enhanced renal and systemic Th1 response. Although both studies showed differences regarding the effect of Treg depletion on the Th17 response and on mouse anti-sheep antibody titers, both observed an increase of renal effector T cells and macrophages, all primarily located within interstitial and periglomerular areas (Ooi et al. 2011; Paust et al. 2011). In addition, an alternative approach demonstrated that in vivo expansion of endogenous Tregs using IL-2/anti-IL-2 monoclonal antibody (mAb) complexes protected mice from glomerular injury during NTN (Klinge et al. 2019) (Fig. 1).

### ANCA-associated anti-myeloperoxidase GN

Autoimmune disease results from loss of central tolerance to specific autoantigens. In a murine model of ANCA-associated anti-myeloperoxidase (MPO) GN, autoreactive MPO-specific CD4^+^ T cells (Gan et al. 2013) and CD8^+^ T cells (Chang et al. 2017) were shown to mediate glomerular injury. In this model, depletion of Tregs by anti-CD25 mAb enhanced the frequencies of MPO-specific IFNγ- and IL-17A-expressing T cells and aggravated glomerular disease, thereby demonstrating the importance of Tregs in the maintenance of tolerance against the MPO autoantigen (Tan et al. 2013) (Fig. 1).

### Anti-glomerular basement membrane GN

Autoimmune diseases are typically associated with specific human leukocyte antigen (HLA) alleles coding for MHCII molecules crucial for antigen presentation. Goodpasture’s disease or anti-GBM GN is an HLA-linked autoimmune renal disorder associated with HLA-DR15 whereas the HLA-DR1 allele was linked with protection (Phelps and Rees 1999). Anti-GBM GN is characterized by CD4^+^ T cells and antibodies directed against the Goodpasture autoantigen α3(IV)NC1 of the GBM (Cairns et al. 2003). In humanized HLA-DR15 transgenic mice (Ooi et al. 2017) or in DBA/1 mice (Hünemörder et al. 2015), immunization with the autoantigen resulted in renal expansion of α3(IV)NC1-specific CD4^+^ T cells that produced IFNγ or IL-17A and mediated development of crescentic GN. In the humanized mouse model, HLA-DR1 co-expression provided immune tolerance. Protection from glomerular disease in HLA-DR15/DR1 double transgenic mice was mediated by autoantigen-specific CD4^+^ Foxp3^+^ Tregs, which expanded upon immunization and secreted tolerogenic cytokines (Ooi et al. 2017). Treg depletion in DBA/1-DEREG mice during immunization aggravated glomerular injury and induced autoantibody production and expansion of autoantigen-specific renal effector CD4^+^ T cells indicating a protective effect of Tregs during development of autoimmunity. However, in vivo expansion of Tregs with IL-2/anti-IL-2 mAb complexes in DBA/1-DEREG mice with established anti-GBM GN failed to prevent loss of kidney function and autoantibody titers were even increased (Klinge et al. 2019) (Fig. 1).

This observation might be explained by an impaired Treg function during chronic stages of anti-GBM GN. Tregs are a highly heterogeneous T cell population whose stability is regulated by certain surface molecules, cytokines and other environmental factors (Min 2017). For example, Treg stability depends on the local cytokine milieu. While IL-2 and its induced phosphorylation of the transcription factor STAT5 were shown to be essential for Treg stability and proliferation, TNFα decreased Foxp3 phosphorylation and thereby destabilized Tregs (Nie et al. 2013). In addition, in the presence of inflammatory cytokines, Foxp3^+^ Tregs were found to express IL-17 and the Th17-associated transcription factor RORγt. This plasticity was accompanied by a reversible loss of immunosuppressive function depending on the stimulus (Beriou et al. 2009). Moreover, the methylation status of the Foxp3 promoter (Floess et al. 2007; Polansky et al. 2008), polyubiquitination and degradation of Foxp3 (Chen et al. 2013) and expression levels of anti-apoptotic Bcl*-2* (Wang et al. 2012) have been identified to affect Treg stability. Thus, identification of mechanisms responsible for loss of tolerogenic Treg function during anti-GBM GN might unravel targets for future therapies.

### Systemic lupus erythematosus

SLE is a systemic autoimmune disease, which is frequently associated with GN, named lupus nephritis. SLE results from a breakdown of tolerance to ubiquitous nuclear antigens including double-stranded DNA (Rahman and Isenberg 2008). IL-17-producing T cells have been associated with the disease in patients and lupus-prone mice (Crispín and Tsokos 2010). Moreover, impaired IL-2 production was observed in SLE patients (Linker-Israeli et al. 1983) and lupus-prone mice (Dauphinée et al. 1981). Pathogenicity of IL-2 deficiency was proven by infection of MRL/lpr mice with a vaccinia recombinant virus-based vector system expressing the IL-2 gene leading to prolonged survival, decreased autoantibody titers, as well as attenuated kidney interstitial infiltration and intraglomerular proliferation (Gutierrez-Ramos et al. 1990).

Peripheral Foxp3^+^ Treg survival and proliferation strictly depend on IL-2 (Fontenot et al. 2005; Setoguchi et al. 2005) and absence of IL-2 signaling in mice causes autoimmune disorders (Malek et al. 2002; Setoguchi et al. 2005). Studies in lupus-prone New Zealand Black × New Zealand White (NZB/W) F1 mice revealed that the ratio of CD4^+^ Foxp3^+^ Tregs to CD4^+^ Foxp3^−^ conventional T cells declined in lymphoid organs of these animals. Further, the percentage of Tregs expressing the IL-2 receptor CD25 decreased with advancing disease, thereby resembling the phenotype of Tregs in IL-2-deficient mice. Interestingly, Tregs from NZB/W F1 mice were functionally intact and IL-2 treatment, which favored Treg proliferation compared to conventional T cells, improved proteinuria and survival (Humrich et al. 2010). In another study, NZB/W F1 mice were treated with the IL-2/anti-IL-2 mAb complex, which decreased the frequencies of renal and splenic IFNγ^+^ and IL-17A^+^ CD4^+^ T cells, reduced autoantibody levels and attenuated glomerular and tubular injury (Yan et al. 2017). Likewise, lupus-prone MRL/lpr mice also featured IL-2 deficiency and restored IL-2 production ameliorated GN (Song et al. 2010). In summary, these studies demonstrated the importance of IL-2 for a functional Treg compartment that ensures tolerance against autoantigens driving SLE (Fig. 1).

In SLE patients, elevated levels of IL-23 were shown, a cytokine that induced IL-17 production in patient-derived T cells while IL-2 expression was limited. Interestingly, IL-23 receptor-deficient MRL/lpr mice displayed attenuated lupus nephritis associated with an increased expression of IL-2 while IL-17 was reduced (Dai et al. 2017). Hence, blockade of IL-23 will not only downregulate the inflammatory IL-17 response but could also increase IL-2 signaling and thereby potentially Treg expansion in patients with SLE.

### Heterogeneity of renal Tregs in immune-mediated GN

A frequent observation is that Tregs from nephritic mice are equally or even more suppressive compared to those from naïve animals (Ooi et al. 2011; Paust et al. 2011). A possible explanation is that Tregs change their phenotype depending on the local cytokine milieu, expand and infiltrate into the inflamed organ to specifically suppress the corresponding local pro-inflammatory T helper (Th) cell response (Krebs and Steinmetz 2016). For example, in a co-culture system of CD4^+^ Foxp3^−^ responder T cells and Foxp3^+^ Tregs, those isolated from lymph nodes of nephritic NTN mice produced more of the anti-inflammatory cytokine IL-10 and showed an increased capacity to suppress responder T cell proliferation and production of pro-inflammatory cytokines compared to Tregs from naïve animals (Ooi et al. 2011).

Recently, a series of studies provided evidence for the existence of specialized Treg subsets, which express a Th subtype-specific transcription factor in addition to Foxp3 and suppress the corresponding Th cell response (Krebs and Steinmetz 2016). During Th1 inflammation, IFNγ was shown to upregulate the Th1 transcription factor T-bet and the chemokine receptor CXCR3 in Tregs, thereby supporting homeostasis, migration and function of Tregs under inflammatory conditions to ensure proper control of Th1 immunity (Koch et al. 2009). Moreover, CXCR3-deficient Tregs failed to control Th1-mediated liver inflammation because of impaired organ infiltration but without losing their immunosuppressive capacity (Erhardt et al. 2011). In NTN, T-bet^+^ Foxp3^+^ Tregs (Treg1) were shown to accumulate in kidneys of nephritic mice. A Treg-specific knockout of T-bet in Foxp3^Cre^xT-bet^fl/fl^ mice resulted in an increased renal Th1 immunity, while the IL-17 response remained unchanged and aggravated NTN. Again, in vitro suppression assays demonstrated intact function of Treg1. However, reduced frequencies of Tregs were detectable in kidneys of Foxp3^Cre^xT-bet^fl/fl^ mice indicating impaired trafficking of T-bet-deficient Tregs most likely due to lack of CXCR3 expression (Nosko et al. 2017). Likewise, renal Treg infiltration was compromised in Foxp3^Cre^xCXCR3^fl/fl^ mice (Paust et al. 2016), which also developed more severe NTN, demonstrating the importance of CXCR3 expression on Th1 subtype-specific Tregs to control renal Th1 immunity.

Subtype-specific Tregs also regulate Th17 immune responses without affecting Th1 immunity. Foxp3^+^ Treg17 cells express one of the master transcription factors of Th17 cells, STAT3, as well as the Th17 cell-specific chemokine receptor CCR6. Foxp3^Cre^xStat3^fl/fl^ mice, which lack Treg17 cells, were more susceptible towards crescentic GN in the NTN model (Kluger et al. 2014a) and towards SLE in a mouse model inducible by pristine (Kluger et al. 2016a). Treg17-deficient animals showed impaired renal trafficking of Tregs and increased Th17 responses (Kluger et al. 2014a, 2016a). Foxp3^+^ Tregs can also express RORγt (Lochner et al. 2008), the second master transcription factor critical for development of Th17 cells. These cells, called biTregs, rapidly expanded in kidney and spleen during NTN (Kluger et al. 2016b). In contrast to Treg17 cells, which do not express IL-17 (Kluger et al. 2014a), biTregs produced IL-17 in addition to the anti-inflammatory cytokine IL-10 (Osorio et al. 2008; Kluger et al. 2016b). BiTregs were present in Treg17-deficient mice and constitute an independent, bifunctional Treg subset. In line with this, adoptive transfer of biTregs suppressed NTN, while cell-specific deletion of RORγt in Foxp3^Cre^xRorc^fl/fl^ mice resulted in significant protection from NTN, indicating a pro-inflammatory role of endogenous biTregs most likely due to their production of IL-17 (Kluger et al. 2016b). A detailed description of these Th subtype-specific Tregs is given elsewhere in this volume.

## Immune regulation of acute and chronic nephrotoxicity by regulatory T cells

Besides their well-defined role as regulators of immune-mediated kidney injury, Tregs also suppress nephrotoxicity due to toxic organ damage. Nephrotoxicity can be induced by the chemotherapeutic agents cisplatin and adriamycin. Moreover, a common complication of kidney transplantation is renal ischemia–reperfusion injury (IRI), which develops in response to restoration of blood flow and reoxygenation of the previously ischemic tissue and may induce acute kidney injury (AKI). These agents or insults do not primarily activate the immune system. Rather, they induce necrotic cell death of renal tissue cells, which secondarily results in activation of the innate immune response. In addition, AKI can be associated with sepsis, the systemic inflammatory response to infection, which is still a challenge in intensive care units. Sepsis- or toxicity-related sterile inflammation is mediated by pathogen- or damage-associated molecular pattern released by dying cells, pattern-recognition receptors such as toll-like receptors, inflammasome activation, inflammatory cytokine and chemokine expression which induce activation and accumulation of neutrophils and mononuclear phagocytic cells into renal tissue (Hutton et al. 2016; Anders 2016). However, the adaptive immune system, although not activated by specific autoantigens during sterile inflammation, is also critically involved in AKI, as mice deficient in T cells, particularly in CD4^+^ T cells, were shown to be protected from post-ischemic renal injury (Burne et al. 2001; Jang and Rabb 2015). Similar results were obtained for cisplatin nephrotoxicity (Liu et al. 2006). Hence, it seems likely that Tregs regulate the corresponding effector CD4^+^ T cells via their bystander activity probably induced by a local cytokine milieu.

### Renal ischemia–reperfusion injury

**Fig. 2:**
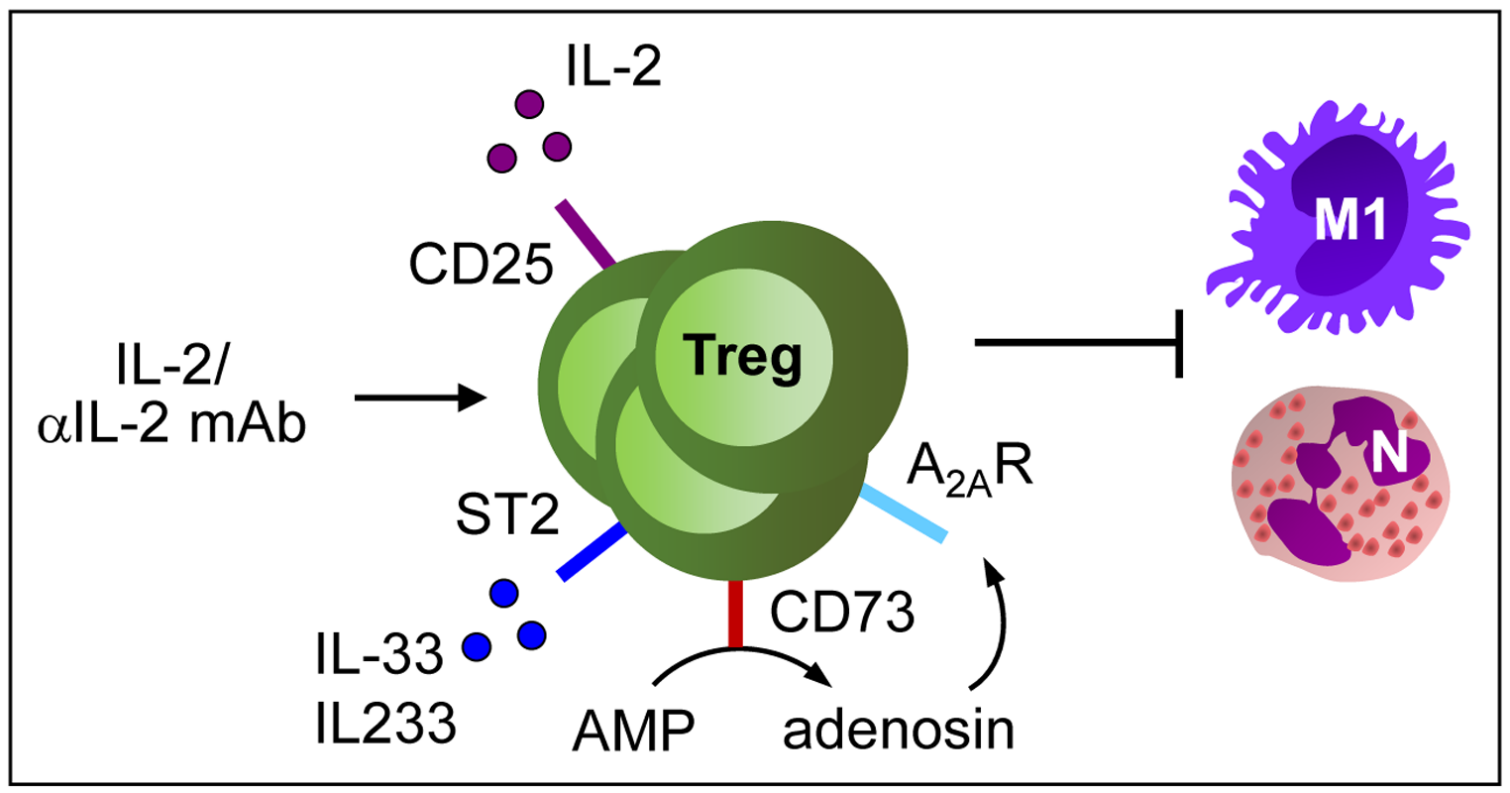
Mechanisms of Treg activation in acute and chronic nephrotoxicity. Application of the IL-2/anti-IL-2 mAb complex as well as the cytokines IL-2, IL-33 or IL-233 induce c and expansion and protect against tissue damage in acute and chronic nephrotoxicity by inhibiting innate immune responses. N: neutrophils, M1: M1 macrophages

The role of CD4^+^ CD25^+^ Foxp3^+^ Tregs for IRI in mice has been demonstrated by depletion of these cells using anti-CD25 mAb PC61, which aggravates the severity of tubular necrosis, kidney damage and inflammation (Kinsey et al. 2009). In another approach, *Rag1*^*−/−*^ mice, which lack T and B cells, were reconstituted with lymph node (LN) cells from either wild-type or scurfy mice, which lack Foxp3 expression and therefore Foxp3^+^ Tregs and then subjected to IRI. Exacerbation of renal IRI in *Rag1*^*−/−*^ mice was more pronounced following adoptive transfer of LN cells from scurfy mice compared to those from wild-type animals. Co-transfer of scurfy LN cells together with wild-type Tregs blunted this effect. Treg transfer before induction of IRI prevented accumulation of neutrophils, macrophages and CD4^+^ T cells in the kidney during reperfusion. Interestingly, adoptive transfer of wild-type Tregs protected *Rag1*^*−/−*^ mice from mild kidney IRI and inhibited renal neutrophil accumulation, demonstrating that Tregs suppress innate immune cells in absence of adaptive immunity (Kinsey et al. 2009). In a therapeutic approach, the IL-2/anti-IL-2 mAb complex was administered to wild-type mice, which expanded Foxp3^+^ Tregs in kidney and spleen, inhibited renal inflammation and damage in response to reperfusion injury and was also effective upon curative treatment (Kim et al. 2013) (Fig. 2).

Interestingly, IL-2 upregulates expression of the IL-33 receptor ST2 on CD4^+^ T cells (Sharma et al. 2011). IL-33 is an alarmin, which is induced in the kidney in response to renal IRI and seems to be involved in IRI pathophysiology (Liang et al. 2017; Ferhat et al. 2018). Moreover, IL-33 has been shown to support Treg recruitment and stability during inflammation (Schiering et al. 2014) and ST2^+^ Tregs, also called “repair” Tregs, were found to regulate local immune responses to tissue injury (Panduro et al. 2016; Zhang et al. 2017a, b). As discussed before, IL-2 is crucial for Treg stability and therefore, it was hypothesized that IL-2 and IL-33 might cooperate to enhance Treg function in the kidney in response to renal IRI (Stremska et al. 2017). Indeed, co-injection of IL-2 and IL-33 significantly increased expansion of Tregs in blood and spleen in contrast to IL-2 or IL-33 treatment alone and protected mice from renal IRI. The authors generated a novel hybrid cytokine, IL-233, bearing IL-2 and IL-33 activities in a single molecule, which was even more effective against IRI than co-application of IL-2 and IL-33. The hybrid cytokine increased Treg numbers also in the kidney, in particular in response to IRI. Interestingly, IL-233 induced a shift of Treg numbers from spleen to kidney in response to IRI, suggesting that IL-233 enhanced the mobilization of Tregs into the inflamed organ. Moreover, administration of the hybrid cytokine to mice increased the suppressive function of splenic Tregs in vitro in a suppression assay and in vivo against renal IRI in adoptive transfer experiments (Stremska et al. 2017). Recently, immune cell landscaping of Tregs isolated from kidneys after IRI revealed a tissue-resident Treg signature characterized by gene expression of ST2, amphiregulin (AREG) CTLA-4 and KLRG1. Pre-treatment of mice with IL-2/anti-IL-2 mAb complex/IL-33 strongly expanded renal Tregs and protected against tissue damage and development of fibrosis. Further, transcriptional profiling of Tregs derived from mice after AKI or during renal fibrosis showed differential expression of genes associated with regeneration and angiogenesis or hyperactivation and fibrosis depending on the inflammatory environment (Do Valle Duraes et al. 2020) (Fig. 2).

### Drug-induced nephrotoxic injury

One dose-limiting toxicity of the chemotherapeutic drug cisplatin is induction of AKI whereas adriamycin induces chronic kidney disease (CKD) in rodent models. In both cases, nephrotoxicity is associated with production of pro-inflammatory cytokines and activation of innate immune cells such as neutrophils and macrophages (Manohar and Leung 2018; Lee and Harris 2011). Both wild-type and athymic nude (nu/nu) mice, which lack mature T cells, were sensitive towards cisplatin nephrotoxicity, although nu/nu mice showed a better overall survival (Lee et al. 2010). Adoptive transfer of CD4^+^ CD25^+^ Tregs protected nu/nu mice from cisplatin-induced renal injury by inhibition of TNFα and IL-1β production as well as macrophage accumulation in the kidney. Likewise, depletion of CD4^+^ CD25^+^ Tregs by anti-CD25 mAb aggravated kidney damage in wild-type mice (Lee et al. 2010). Hence, similar to AKI induced by IRI, CD4^+^ CD25^+^ Tregs suppressed acute nephrotoxicity in the absence of adaptive immunity. Moreover, pretreatment of mice with the hybrid cytokine IL-233, which increased ST2-expressing Tregs, protected mice from cisplatin-induced nephrotoxicity (Stremska et al. 2017) (Fig. 2).

Similar studies were performed in the mouse model of adriamycin-induced CKD where T and B cell-deficient severe combined immunodeficiency (scid) mice were protected from glomerular and interstitial injury following reconstitution with CD4^+^ CD25^+^ Tregs (Mahajan et al. 2006). Moreover, adoptive transfer of Foxp3-transduced CD4^+^ T cells prevented adriamycin nephropathy and interstitial accumulation of T cells and macrophages (Wang et al. 2006). In addition, Treg expansion induced by administration of the IL-2/anti-IL-2 mAb complex (Polhill et al. 2012) or IL-233 (Stremska et al. 2017; Sabapathy et al. 2019) protected mice from CKD. IL-233 treatment preserved renal function and fibrosis when given either before or even at very late time points after induction of CKD, which correlated with induction of reparative mechanisms in kidneys of adriamycin-treated mice. The protective effect of IL-233 regarding renal inflammation, injury and fibrosis was blunted by administration of anti-CD25 mAb PC61, which depleted Tregs in spleen but not in kidneys (Sabapathy et al. 2019) (Fig. 2).

## Mechanisms of Treg-mediated immune regulation in renal inflammation

Multiple mechanisms have been proposed for Treg-mediated immunosuppression including contact-dependent mechanisms and soluble factors. These involve the cell surface molecules CTLA-4, CD25, PD-L1, TIGIT, LAG-3 TIM-3 and CD73/CD39, the cytokines IL-10, TGFβ and IL-35, as well as adenosine, cyclic (c)AMP and the cytotoxic molecule granzyme B (detailed reviewed in Shevach 2009; Josefowicz et al. 2012). Treg-dependent mechanisms of renal immunosuppression were widely studied in models of immune-mediated GN and the molecules most frequently investigated were anti-inflammatory cytokine IL-10 and the co-inhibitory molecule programmed-death ligand (PD-L)1.

### IL-10

One important player of Treg-mediated immunosuppression is the anti-inflammatory cytokine IL-10, which is released from Tregs to suppress immune responses and therefore, might protect against autoimmunity. First evidence for a role of IL-10 in renal immune regulation derived from IL-10-deficient mice that showed an elevated Th1 response and developed more severe NTN (Kitching et al. 2000). However, the cellular source of IL-10 remained still elusive. To detect IL-10-producing cell populations in kidneys of nephritic NTN mice, we used double-knockin reporter mice, which allowed detection of IL-10/GFP as well as Foxp3/mRFP (FIR x tiger mice) (Ostmann et al. 2013). Seven days after NTN induction, we determined a significant increase of IL-10^+^ Foxp3^+^ Tregs in kidneys of nephritic FIR x tiger mice compared to healthy controls. Moreover, elevated frequencies of renal IL-10-expressing Th cells, DCs, macrophages and B cells were also detected in nephritic FIR x tiger mice, indicating that other cell populations besides Tregs produced IL-10 in response to renal inflammation. To analyze the role of endogenous IL-10^+^ Tregs during NTN, we generated Foxp3^Cre^xIL-10^fl/fl^ mice. These mice developed more severe NTN than control mice assessed by increased glomerular crescent formation and albuminuria and an elevated Th17 immune response. However, Treg frequencies remained unaltered in inflamed kidneys of Foxp3^Cre^xIL-10^fl/fl^ mice. The importance of Treg-derived IL-10 was further confirmed by adoptive transfer experiments with Tregs from IL-10-deficient mice, which failed to prevent NTN in contrast to wild-type Tregs (Ostmann et al. 2013). IL-10-deficient Tregs did also not protect from renal IRI in *Rag1*^*−/−*^ mice (Kinsey et al. 2009), here by insufficient suppression of the innate rather than adaptive immunity (Fig. 1).

Foxp3^Cre^xIL-10Ra^fl/fl^ mice developed a spontaneous hyper-Th17 immune response compared to control mice. Following NTN induction, systemic and renal Th17 immunity was further boosted and crescentic GN as well as renal immune cell infiltration were aggravated. While Treg trafficking remained unaffected in the knockout animals, Tregs from Foxp3^Cre^xIL-10Ra^fl/fl^ mice showed a reduced ability to suppress effector T cell proliferation and IL-17 production in vitro. Moreover, IL-10Ra-deficient Tregs released less IL-10 in vitro and in vivo compared to respective controls (Diefenhardt et al. 2018). Hence, this study provided evidence for a feed-forward loop, in which IL-10Ra signaling mediates IL-10 secretion by Tregs that controls the renal Th17 immune response during NTN (Fig. 1).

As mentioned above, also CD19^+^ B cells produced IL-10 in response to NTN (Ostmann et al. 2013). Indeed, IL-10^+^ regulatory B cells have been implicated in the control of inflammation (Mauri and Bosma 2012). However, pathology of NTN was comparable in CD19^Cre^xIL-10^fl/fl^ and control mice (Kluger et al. 2014b). Along this line, IL-10-producing B cells were unable to prevent the clinical course of lupus nephritis in MRL/lpr mice (Teichmann et al. 2012), indicating that B cell-derived IL-10 has no immunosuppressive function in GN.

### Co-inhibitory pathways

Co-inhibitory pathways induce inhibitory signals in T cells and are crucial for the maintenance of peripheral tolerance. One negative co-stimulator receptor expressed on activated T cells is programed cell death-1 (PD-1), which binds to its ligands PD-L1 and PD-L2. While PD-L2 is exclusively expressed by hematopoietic cells such as activated DCs and macrophages, PD-L1 is expressed by hematopoietic and non-hematopoietic cells and can be further induced by pro-inflammatory cytokines such as IFNγ (Keir et al. 2008). Recently, inappropriate expression of PD-L1 by tumor cells has been recognized as a target for cancer treatment, which resulted in clinical development of immune checkpoint inhibitors to fight cancer immune tolerance (Okazaki and Honjo 2007). The PD-1/PD-L1 pathway mediates immune regulation and tolerance either by direct inactivation of effector T cells (Freeman et al. 2000) or by induction and maintenance of peripheral Tregs (Francisco et al. 2009).

The PD-1/PD-L1 pathway has been implicated in immune regulation of renal diseases. A single nucleotide polymorphism in the PD-1 gene was associated with increased susceptibility of patients towards SLE (Prokunina et al. 2002). Studies in mice revealed that aged PD-1-deficient mice develop lupus-like GN (Nishimura et al. 1999). Moreover, in experimental autoimmune GN in rats, stimulation of the PD-1/PD-L1 pathway with a PD-L1/Fc fusion protein reduced kidney disease and renal inflammatory cell infiltration (Reynolds et al. 2012). Likewise, activation of the PD-1/PD-L1 axis using a PD-L1-Ig attenuated the pro-inflammatory cytokine response and renal injury in lupus-prone mice (Liao et al. 2017). On the other hand, inhibition of the PD-1/PD-L1 pathway using an anti-PD-L1 antibody aggravated lupus nephritis in NZB/W F1 mice and increased their mortality rate. However, antibody-mediated blockade of PD-1 prevented lupus nephritis, probably by depleting CD4^+^ PD-1^high^ T cells that released high amounts of IFNγ (Kasagi et al. 2010). Anti-PD-1 mAb treatment also increased renal macrophage infiltration and worsened glomerular and tubulointerstitial injury and renal dysfunction in adriamycin-induced CKD (Qin et al. 2006). Further, administration of PD-L1- or PD-L2-blocking mAbs and genetic deficiency of either PD-1 ligand exacerbated inflammation, renal function and tubular necrosis in the model of kidney IRI. In this study, experiments using bone marrow chimeric mice revealed that PD-L1 expressed on non-hematopoietic cells was critical for resistance to AKI. In addition, neutralization of either PD-1 ligand blunted the protective effect of adoptively transferred Tregs towards kidney IRI (Jaworska et al. 2015). Accordingly, in the NTN model, mice lacking either PD-L1 or PD-L2 showed increased renal pathology, loss of renal function and inflammatory leukocyte infiltrates. Here, PD-L1 expression on hematopoietic but not on parenchymal cells was responsible for aggravation of NTN (Menke et al. 2007). However, the specific PD-L1-expressing immune cell subtype that mediates the protective effect of PD-L1 in NTN remains to be determined. Since the PD-1/PD-L1 pathway contributes to Treg-mediated control of inflammation by induction and maintenance of Tregs (Francisco et al. 2009), we asked whether Tregs might be responsible for PD-L1-mediated protection in NTN. Interestingly, frequencies of PD-L1^+^ and PD-1^+^ Foxp3^+^ Tregs increased in kidneys of nephritic mice, suggesting a function of the PD-1/PD-L1 pathway in Treg-mediated immune regulation in NTN (Neumann et al. 2019). To investigate the Treg response in absence of PD-1/PD-L1 signaling, we used PD-L1-deficient (*Cd274*^*−/−*^) mice. PD-L1 was shown to be involved in T-cell development (Keir et al. 2005) and Treg induction (Francisco et al. 2009). However, we observed that *Cd274*^*−/−*^ mice showed similar frequencies of Helios^+^ and neuropilin-1^+^ Foxp3^+^ Tregs compared to wild-type mice, thereby excluding developmental defects of thymus-derived Tregs in *Cd274*^*−/−*^ mice. Moreover, we detected increased frequencies of Foxp3^+^ Tregs in kidneys of nephritic *Cd274*^*−/−*^ mice, demonstrating that neither Treg proliferation nor Treg infiltration into the inflamed kidney were compromised in the absence of PD-1/PD-L1 signaling. However, analysis of renal and systemic immunity revealed that the Th1 response but not the Th17 response, was up-regulated in absence of PD-L1, which resulted in more severe NTN assessed by enhanced crescent formation and albuminuria. In order to rule out any influence of the genetic deletion of *Cd274* on renal inflammation and injury, we blocked PD-L1 in NTN-treated wild-type mice using a specific mAb and obtained similar results as observed in nephritic *Cd274*^*−/−*^ mice. Hence, genetic deletion or mAb-mediated blockade of PD-L1 increased Th1 immunity and aggravated NTN despite elevated frequencies of Foxp3^+^ Tregs in the inflamed kidney. Therefore, we further analyzed the phenotype and function of PD-L1-deficient Foxp3^+^ Tregs in homeostasis and renal inflammation. Gene expression profiling of FACS-sorted Foxp3^+^ Tregs isolated from naïve and NTN-treated Foxp3-reporter mice (FIR x tiger and FIR x tiger x *Cd274*^*−/−*^ mice) revealed a distinct gene expression profile of PD-L1-negative Tregs compared to PD-L1-expressing Tregs even in homeostasis. Most importantly, in nephritic mice, PD-L1^−^ Tregs showed decreased expression of genes related to Treg function such as *Il10* and *GzmB* that were otherwise increased in response to kidney inflammation. Moreover, gene expression of the high affinity IL-2 receptor CD25 was maintained at high levels in PD-L1^+^ Tregs from nephritic mice but was down-regulated in PD-L1^−^ Tregs, indicating instability of PD-L1-deficient Tregs under inflammatory conditions. Functional analysis of the immunosuppressive activity of Tregs from nephritic *Cd274*^−/−^ and wild-type mice revealed a decreased capacity of PD-L1^−^ Tregs to suppress effector T cell proliferation in vitro and an inability to prevent NTN upon adoptive transfer in vivo. Hence, Tregs exhibited an impaired immunosuppressive function during NTN in the absence of PD-L1 (Neumann et al. 2019) (Fig. 1).

Like the PD-1/PD-L1 pathway, T-cell immunoglobulin and mucin protein-3 (Tim3) and its ligand galectin-9 (Gal-9) negatively regulate pro-inflammatory immune responses (Chen and Flies 2013). Tim-3 was shown to exert a protective function in NTN since administration of a blocking anti-Tim-3 mAb led to increased renal T cell and macrophage infiltration and aggravated NTN (Schroll et al. 2010). Furthermore, Gal-9 treatment reduced renal infiltration of Th1 and Th17 cells in anti-GBM GN and ameliorated renal injury (Zhang et al. 2014), indicating that in addition to PD-1/PD-L1, other co-inhibitory pathways may also regulate crescentic GN.

### CD39/CD73 and adenosine

Tregs highly express the cell surface ectonucleotidases CD39 and CD73 to synthesize the anti-inflammatory mediator adenosine by degradation of extracellular ATP, which is a pro-inflammatory danger signal released by dying cells during tissue injury. CD39 and CD73 sequentially dephosphorylate extracellular ATP into its metabolites ADP and AMP (CD39) and AMP into adenosine (CD73). Adenosine binds to its receptor A_2A_R expressed by immune effector cells, which results in anti-inflammatory signaling by elevation of intracellular cAMP levels (de Oliveira Bravo et al. 2016; Trautmann 2009).

The anti-inflammatory potency of A_2A_R agonists has been analyzed in the kidney where they prevented renal IRI (Okusa et al. 1999). Likewise, an antagonist for the pro-inflammatory ATP receptor P2X7 has been shown to ameliorate kidney dysfunction and inflammation in renal IRI. Interestingly, the protective effect of the P2X7 antagonist was mediated via Treg expansion since depletion of these cells using the anti-CD25 mAb PC61 abrogated the beneficial effect of the antagonist (Koo et al. 2017). One study analyzed the role of CD73 and A_2A_R expressed by Tregs for renal damage in kidney IRI. In contrast to adoptively transferred wild-type Tregs, CD73-deficient or A_2A_R-deficient Tregs failed to protect mice from IRI-induced renal damage (Kinsey et al. 2012). Therefore, Treg-generated adenosine may act on innate immune cell activation induced by IRI and in an autocrine fashion on the Tregs themselves (Fig. 2). The mechanism of this feedback loop seemed to depend on A_2A_R-dependent enhanced surface expression of PD-1 on Tregs since their protective effect upon adoptive transfer was inhibited by pre-incubation with a blocking anti-PD-1 mAb. Thus, both the ability to generate and to respond to adenosine was required for Tregs to suppress kidney IRI through a PD-1-dependent mechanism (Kinsey et al. 2012). In contrast, by using reciprocal bone marrow chimera of CD73-deficient and wild-type mice, it has been demonstrated that parenchymal but not hematopoietic expression of CD73 was responsible for inhibition of kidney IRI since *Cd73*^*−/−*^ recipients showed elevated levels of plasma creatinine and more severe tubular damage compared to wild-type recipients. These results were substantiated by showing that mice with a specific deletion of CD73 in proximal tubular epithelial cells (PTECs) were more sensitive towards renal inflammation and injury compared to several other cell-specific CD73 knockouts, indicating that CD73 expressed by PTECs is crucial for protection from kidney IRI (Sung et al. 2017).

## Renal iTreg induction by tolerogenic DCs and M2 macrophages

Besides thymus-derived nTregs also iTregs, which differentiate from naïve Foxp3^−^ CD4^+^ T cells in the periphery, are crucial for the maintenance of peripheral tolerance and for immunosuppression during inflammation. Two subsets of APCs, namely tolerogenic DCs and M2 macrophages, have been described to be important for iTreg induction. Tolerogenic DCs are characterized by low expression of MHCII and co-stimulatory molecules, which has been shown to favor iTreg induction (Apostolou et al. 2004; Kretschmer et al. 2005; Domogalla et al. 2017). Tolerogenic DCs were found to induce iTregs by production of IL-10 (Akbari et al. 2001; Wakkach et al. 2003; Levings et al. 2005) or TGFβ (Travis et al. 2007). There are different subsets of DCs identified that promote iTreg development in secondary lymphoid organs including plasmacytoid DCs and CD103^+^ DCs (Ochando et al. 2006; Irla et al. 2010; Coombes et al. 2007).

In the kidney, a subset of basic leucine zipper ATF-like transcriptional factor 3 (Batf3)-dependent CD103^+^ DCs was found to be crucial for renal iTreg induction and accumulation in crescentic GN. Absence of this DC subset in mice lacking Batf3 resulted in reduced renal Treg numbers and an aggravated course of GN (Evers et al. 2016). Several factors promote the tolerogenic phenotype of DCs and thus, iTreg induction, including IL-10 (Tai et al. 2011), vitamin D (Ferreira et al. 2014), or inhibition of nuclear factor κB (NFκB) (Iruretagoyena et al. 2006). In a therapeutic approach, adoptive transfer of tolerogenic DCs, generated in vitro in presence of a NFκB inhibitor and pulsed with the antigen MPO, decreased established antigen-specific anti-MPO T-cell immunity and glomerular injury in the mouse model of autoimmune anti-MPO GN. Mechanistically, tolerogenic DCs induced IL-10-producing Tregs in MPO-immunized mice. In co-culture experiments, the tolerogenic DCs generated IL-10^+^ Foxp3^+^ Tregs from CD4^+^ Foxp3^−^ T cells via inducible costimulator (ICOS). The protective effect of tolerogenic DCs on anti-MPO GN was blunted when CD4^+^ Foxp3^+^ Tregs were depleted in vivo. When Tregs, expanded by tolerogenic DCs in vitro, were adoptively transferred into mice with anti-MPO GN, they suppressed anti-MPO immunity and GN via IL-10, as demonstrated by neutralization experiments with an anti-IL-10 receptor antibody. The authors suggested that autoantigen-loaded tolerogenic DCs might be a novel treatment option for anti-MPO GN (Odobasic et al. 2019).

Alternatively activated M2 macrophages are characterized by expression of the mannose receptor and the anti-inflammatory cytokine IL-10. M2 macrophage polarization occurs in response to various stimuli including the cytokines IL-4, IL-13, IL-10 and TGFβ (Gordon Immunity 2010). Human M2 macrophages, generated in vitro in presence of IL-4/IL-10/TGFβ, were shown to induce a TGFβ-dependent differentiation of Foxp3^+^ Tregs from naïve CD4^+^ T cells (Schmidt et al. 2016). In another study, M-CSF-induced M2 macrophages favored iTreg induction, which mediated their immunosuppressive function by expression of TGFβ (Savage et al. 2008).

In general, renal macrophages have been described to exacerbate kidney injury (Nikolic-Paterson et al. 2001; Schlondorff et al. 2008). However, there is some evidence that M2 macrophages have beneficial effects on disease pathology. Adoptive transfer of IL-4/IL-13-polarized M2 macrophages in SCID mice with adriamycin nephropathy was shown to improve kidney function potentially by inhibiting inflammatory macrophage infiltration (Wang et al. 2007). Transfer of in vitro polarized M2 macrophages also suppressed development of interstitial fibrosis in murine diabetes-induced renal injury (Zheng et al. 2011). Further, IL-25-mediated M2 macrophage polarization in vivo reduced proteinuria and glomerulosclerosis in adriamycin-induced CKD (Cao et al. 2011). During IRI, deletion of IL-4 and IL-13 (Zhang et al. 2017a, b) or proximal tubule-derived CSF-1 (Wang et al. 2015) resulted in reduced M2 polarization and inhibited recovery from acute kidney injury.

Regarding M2 macrophage-mediated iTreg induction in kidney disease, there is one study describing that in kidney transplantation, preoperative transfer of donor-derived regulatory macrophages (Mregs), which have been polarized in vitro in presence of M-CSF and IFNɣ, resulted in increased numbers of circulating TIGIT^+^ Foxp3^+^ Tregs in living-donor kidney transplant recipients. The authors suggested a feed-forward mechanism by which Mregs might promote allograft survival by iTreg induction (Riquelme et al. 2018). Moreover, adoptively transferred IL-10/TGFβ-polarized M2 macrophages were shown to attenuate renal inflammation and decline in kidney function in murine adriamycin nephropathy. The M2 macrophages promoted differentiation of iTregs from naïve CD4^+^ T cells in vitro and increased the number of Tregs in draining lymph nodes in vivo (Cao et al. 2010). In a following study, M2 macrophages were subdivided into IL-4/IL-13-polarized M2a and IL-10/TGFβ-polarized M2c macrophage subsets. After adoptive transfer, both subsets ameliorated renal inflammation and injury in adriamycin nephropathy. However, M2c macrophages showed a stronger immunosuppressive capacity and more effectively reduced proteinuria and renal fibrosis. This was attributed to the ability of M2c but not M2a macrophages to promote iTreg differentiation in vitro and to enhance Treg numbers in draining lymph nodes in vivo (Lu et al. 2013).

## Regulation of renal inflammation by innate immune cells

### Type 2 innate lymphoid cells

Type 2 innate lymphoid cells (ILC2) are effector cells of the innate immune response, which become rapidly activated during infection and inflammation, e.g., by release of alarmin IL-33 from damaged cells. ILC2 express the transcription factor GATA3 and produce IL-5 and IL-13 as well as the epidermal growth factor AREG. ILC2 are major players in allergy and asthma but also mediate tissue injury, fibrosis and regeneration in other organs (Klose and Artis 2016; Ochel et al. 2019).

**Fig. 3:**
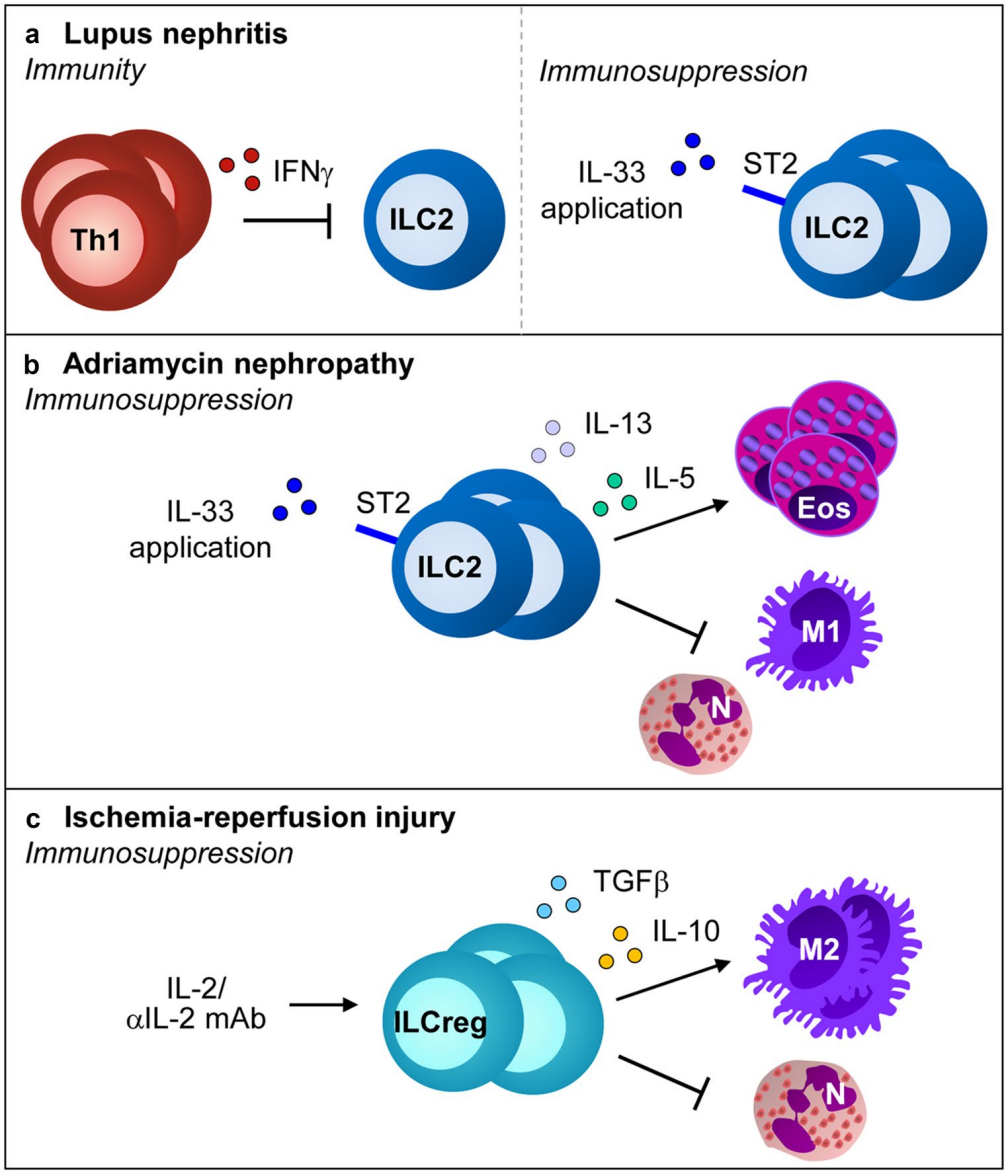
Activation and function of ILC2 in kidney disease. Application of IL-33 induces activation and expansion of renal ILC2 while IFNγ has an inhibitory effect. Activated ILC2 exert an immunosuppressive function in adriamycin nephropathy through inhibition of M1 macrophages and neutrophils as well as activation and recruitment of eosinophils. In IRI, treatment with the IL-2/anti-IL-2 mAb complex induces ILCreg, which promote M2 macrophage polarisation by production of TGFβ and IL-10. N: neutrophils, M1: M1 macrophages, Eos: eosinophils, M2: M2 macrophages

A recent study showed that in MRL/lpr mice, progression of lupus nephritis was accompanied by a continuous reduction of ILC2 numbers in the inflamed kidney, which was most likely mediated by the chronic Th1 immune response. Interestingly, treatment of MRL/lpr mice with the ILC2-activating cytokine IL-33 restored renal ILC2 numbers and attenuated lupus nephritis (Düster et al. 2018). However, mechanisms by which renal ILC2 mediate their immunosuppressive function in SLE remain to be determined. One might speculate that IL-33-activated ILC2 release AREG, which was shown to maintain and enhance Treg function during inflammation (Zaiss et al. 2013). Like IL-33, the hybrid cytokine IL-233 also expanded ILC2 in the kidney (Stremska et al. 2017). ILC2 typically expresses not only ST2 but also CD25 (Roediger et al. 2013). Interestingly, adoptive transfer of ILC2, expanded in vitro by IL-233 (Stremska et al. 2017) or IL-2/IL-7/IL-33 (Cao et al. 2018), protected mice against renal IRI. Moreover, administration of recombinant IL-33 to mice several days before induction of IRI expanded ILC2, Tregs and anti-inflammatory M2 macrophages in the kidney and prevented renal injury. The protective effect of IL-33 was mediated by ILC2 and M2 macrophages but not by Tregs. Interestingly, IL-33 and ILC2 were also protective upon curative treatment administered 24 h after IRI induction (Cao et al. 2018). Along the same line, short-term IL-33 treatment was demonstrated to expand ILC2 and attenuated renal inflammation and glomerulosclerosis in adriamycin nephropathy (Riedel et al. 2017). The functional role of ILC2 for the reno-protective effect was shown in ILC-deficient *Rag2*^*−/−*^* x Il2rcg*^*−/−*^ mice, where IL-33 administration failed to reduce adriamycin-induced inflammation and glomerulosclerosis. Moreover, expression of macrophage markers linked with a regulatory M2 phenotype was shown in IL-33-treated nephritic wild-type mice, which was associated with renal expression of IL-5 and IL-13, a cytokine that induces polarization of M2 macrophages (Gordon 2003). IL-5 instead is a strong activator of eosinophils. Interestingly, IL-33 treatment in the absence of eosinophils, i.e., in eosinophil-deficient ΔdblGATA mice, failed to protect from adriamycin nephropathy despite an unaltered expansion of ILC2 (Riedel et al. 2017). Hence, IL-33-activated ILC2 and their mediators seem to activate a network of immune regulatory and tissue repair cells such as Tregs, M2 macrophages and eosinophils that dampen organ inflammation and favor tissue regeneration (Fig. 3). However, since IL-33 was shown to play a critical role in the pathogenesis of renal IRI (Liang et al. 2017; Ferhat et al. 2018) and drug-induced AKI (Akcay et al. 2011), therapeutic safety of this cytokine should be carefully evaluated.

In a recent paper, it was demonstrated that administration of the IL-2/anti-IL-2 mAb complex expanded regulatory ILC2 (ILCregs). These cells showed immunosuppressive effects on pro-inflammatory ILC1 and macrophages in vitro via production of IL-10 and TGFβ. Likewise, adoptive transfer of ILCregs prevented renal IRI along with a reduction of neutrophil infiltration and induction of M2 macrophages. In addition, the IL-2/anti-IL-2 mAb complex prevented renal IRI in *Rag*^*−/−*^ mice, which was probably due to induction of ILCregs (Cao et al. 2020). These observations may provide a possible explanation of the protective effect of transferred Tregs against renal IRI in *Rag1*^*−/−*^ mice that still harbor ILC2 (Fig. 3). As T cells directly interact with ILC2 either by cytokine production or TCR-MHCII interactions (Gasteiger and Rudensky 2014; Steinmann et al. 2020), Tregs might have polarized and activated ILCregs to suppress the pro-inflammatory innate immune response in *Rag*^*−/−*^ mice, which is raised upon renal IRI.

### Mast cells

Mast cells (MC) are known to promote tissue injury, in particular in the context of allergic disease. However, MC have been shown to accumulate in renal interstitium of patients with various types of GN (Tóth et al. 1999; Ehara and Shigematsu 1998) and were found to play a pathogenic role in the mouse model of NTN (Timoshanko et al. 2006). In contrast, in accelerated NTN, MCs were demonstrated to control the immune response. In MC-deficient mice, an increase of infiltrating interstitial macrophages, CD4^+^ and CD8^+^ T cells compared to MC-proficient mice was observed in NTN and aggravation of inflammation correlated with increased proteinuria and glomerular damage (Hochegger et al. 2005). A follow-up study showed that Treg-derived IL-9, a MC growth and activation factor, induced recruitment of MC into renal draining lymph nodes thereby suppressing immune activation in the kidney during NTN (Eller et al. 2011). A similar immune inhibitory mechanism has been described in a model of transplantation tolerance, where Treg-derived IL-9 recruited and activated MC in tolerant tissue (Lu et al. 2006). However, the subtypes of MC that mediate either inflammation or immune regulation in the kidney have not been identified yet. Recently, a mechanism for MC-mediated immunosuppression has been described, in which MC produce AREG that binds to the EGF receptor expressed by Tregs under inflammatory conditions thereby enhancing their immunosuppressive capacity in vitro and in vivo (Zaiss et al. 2013). Hence, it seems that Tregs and MC amplify their immunosuppressive function by an IL-9- and AREG-dependent autocrine loop. Moreover, in ANCA-associated anti-MPO GN, MCs were shown to ameliorate disease pathology by IL-10 production that ensured Treg recruitment and immunosuppressive function (Gan et al. 2012).

### Proximal tubular epithelial cells

Besides professional APCs like DCs and macrophages, other MHCII-expressing cell populations have been identified, which also exert the capacity to stimulate CD4^+^ T cells and therefore, are termed non-professional APCs. Particularly epithelial and endothelial cells have been suggested to play a role in the initiation and regulation of CD4^+^ T cell responses during inflammation (Kambayashi and Laufer 2014). In the kidney, several studies have demonstrated low MHCII expression by proximal tubular epithelial cells (PTECs) of healthy humans (Gastl et al. 1996) and naïve mice (Hagerty and Allen 1992), which was up-regulated in renal cell carcinoma patients (Gastl et al. 1996) as well as murine lupus nephritis (Wuthrich et al. 1989), graft-versus-host disease (Sinclair et al. 1984) and kidney transplants (Kouwenhoven et al. 2001). Moreover, PTECs were shown to process and present soluble antigens, thereby inducing activation of CD4^+^ T cell hybridoma (Hagerty and Allen 1992). In comparative analysis, we have recently demonstrated that particularly PTECs and not distal tubular epithelial cells express molecules linked with APC function including MHCII, the co-stimulatory molecules CD80/CD86 and the MHCII invariant chain CD74 in naïve and nephritic NTN mice. In patients with ANCA-associated GN, CD86 expression was also restricted to proximal tubules while expression of MHCII and CD74 was detected in both proximal and distal tubules (Breda et al. 2019).

That non-professional APCs can exert immunomodulatory functions beside professional APCs has been particularly demonstrated in the liver. Here, liver sinusoidal endothelial cells (LSECs) were shown to promote Treg induction (Kruse et al. 2009; Carambia et al. 2014), inhibit inflammatory cytokine production (Carambia et al. 2013), induce T-cell anergy (Diehl et al. 2008) and tolerize professional APCs (Schildberg et al. 2008), thereby contributing to hepatic tolerance induction. In contrast, PTECs did not favor induction of Tregs (Breda et al. 2019) and instead were found to induce antigen-specific CD4^+^ T cell activation and pro-inflammatory cytokine expression in vitro (Breda et al. 2019; Waeckerle-Men et al. 2007). However, compared to professional DCs, PTECs induced a rather weak activation of CD4^+^ T cells probably due to low expression levels of MHCII (Breda et al. 2019). In addition, PTECs were shown to regulate activation of T cells by the co-inhibitory molecule PD-L1. Several studies have reported induction of PD-L1 expression in PTECs by the cytokine IFNγ in vitro, which resulted in suppression of T-cell activation and inflammatory cytokine expression (Waeckerle-Men et al. 2007; Starke et al. 2010; Wilkinson et al. 2011). Thus, PTECs can induce pro-inflammatory CD4^+^ T cell activation that might be regulated through inflammation-induced induction of epithelial PD-L1 expression. However, all data regarding PTEC-mediated T cell activation were derived from in vitro experiments and it still remains unclear whether PTECs exert immunomodulatory function in vivo in the course of GN and what effect epithelial cell-mediated T-cell activation has for disease pathology.

## Conclusion and therapeutic outlook

Autoimmune diseases result from a breakdown of tolerance against self-antigens. Antigen-specific nTregs selected in the thymus represent the major cell population that prevents systemic and organ-specific autoimmunity. Contrary, reduction of cell number, stability, or immunosuppressive function of Tregs can evoke autoimmunity. In the kidney, autoimmune GNs have been associated with aberrant Treg responses displaying different mechanisms of dysregulation. For example, the number of Tregs is reduced in patients with lupus nephritis due to impaired T cell-mediated IL-2 production. Thus, therapeutic expansion of endogenous Tregs using low-dose IL-2 has been successfully established. However, this might not be a treatment option for other autoimmune kidney diseases. Therefore, different strategies to more effectively expand endogenous Tregs are currently investigated in animal models of GN including treatment with IL-2/anti-IL-2 mAb complexes, IL-2 and IL-33 or IL-233. These treatment regimens have the obvious advantage to locally expand and stabilize self-reactive Tregs. Interestingly, these treatment strategies also prevent acute and chronic nephrotoxicity, which could be due to immunosuppressive bystander activities of Tregs or Treg-dependent expansion of ILC2, which seem to be suppressive themselves via activation of a network of immune regulatory cells in the kidney that include Tregs and M2 macrophages.

Cellular Treg therapies, where patient-derived Tregs are expanded in vitro by different protocols and re-injected to the patients, have been extensively studied in clinical trials for the control of allo-reactive immune responses in transplant rejection and self-reactive immunity in autoimmune diseases such as type 1 diabetes, Crohn’s disease and autoimmune hepatitis. These trials use autologous polyclonally expanded Tregs mostly isolated from peripheral blood. The expansion protocols involve un-specific TCR stimulation in the presence of IL-2 and sometimes TGFβ and all-trans retinoic acid. Ongoing clinical trials show safety and feasibility of Treg infusion; however, long-term stability of the administered Tregs as well as their trafficking into the affected organ need further investigation (Romano et al. 2019). In the kidney, clinical trials are currently investigating the therapeutic potential of cellular Treg therapies in kidney transplantation (Romano et al. 2019). Based on the findings in mouse models of autoimmune GN demonstrating the necessity of a functional Treg compartment for disease control, cellular Treg therapies should also be addressed in GN patients.

Besides Treg-based therapy, application of tolerogenic DCs is currently being tested in clinical trials for the treatment of allograft rejection and autoimmune diseases such as type 1 diabetes, rheumatoid arthritis, inflammatory bowel disease and multiple sclerosis since these cells not only induce anergy and apoptosis in effector T cells but also mediate iTreg induction in vivo (Obregon et al. 2017; Cauwels et al. 2020). In kidney disease, the protective potential of donor-derived tolerogenic DCs and also Mregs is being explored in renal transplant patients (Obregon et al. 2017; Amodio et al. 2019; Cauwels et al. 2020). Hence, experience gained in this clinical setup might encourage trials using cell-based immunosuppressive therapy as a treatment option for renal autoimmune diseases.
